# Review of species of the genus *Adelurola* Strand, 1928, with a key to species (Hymenoptera, Braconidae, Alysiinae)

**DOI:** 10.3897/zookeys.566.6684

**Published:** 2016-02-18

**Authors:** Francisco Javier Peris-Felipo, Zahra Yari, Cornelis van Achterberg, Ehsan Rakhshani, Sergey A. Belokobylskij

**Affiliations:** 1 Bleichestrasse 15, CH-4058 Basel, Switzerland; 2 Department of Plant Protection, College of Agriculture, University of Zabol, I.R. Iran; 3 Naturalis Biodiversity Center, P.O. Box 9517, 2300 RA Leiden, The Netherlands; Northwest University, College of Life Sciences, Taibai Road, Xi’an, China; 4 Zoological Institute of the Russian Academy of Sciences, St Petersburg, 199034, Russia; Museum and Institute of Zoology of the Polish Academy of Sciences, Wilcza 64, Warszawa 00–679, Poland

**Keywords:** Hymenoptera, Alysiinae, Adelurola, fly endoparasitoid, new record, new combination, redescription, Iran

## Abstract

The alysiine genus *Adelurola* Strand, 1928 (Hymenoptera, Braconidae) is revised. Illustrated re-descriptions and a key to all known species of this genus are given. The following new combination is proposed: *Dapsilarthra eurys* (Chen & Wu, 1994), **comb. n.** *Adelurola amplidens* (Fischer, 1966) and *Adelurola asiatica* Telenga, 1935 are recorded for the first time from Iran and Kyrgyzstan, respectively.

## Introduction

**Figure 1.**
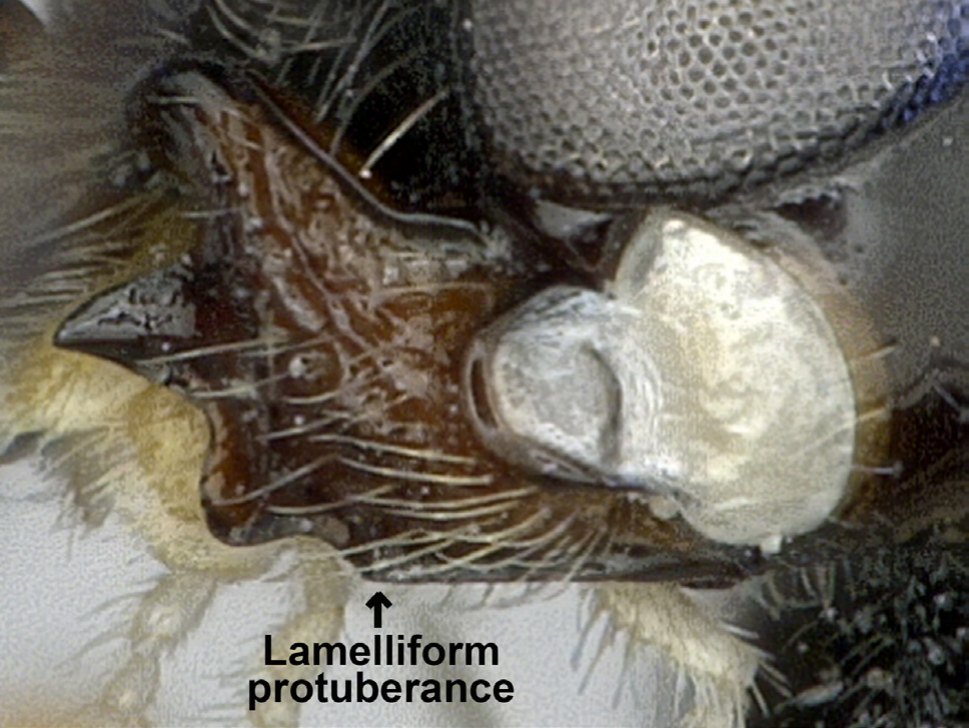
Mandible in *Adelurola*.

*Adelurola* Strand, 1928 is a small Palaearctic genus of the braconid subfamily Alysiinae that currently contains five recognised species (Yu et al. 2012). Traditionally, most species of *Adelurola* were included within *Dapsilarthra* (e.g. Wharton 1980). Van Achterberg (1983) clarified the status of both genera and found valuable differences between them, including the presence of a ventral lamelliform lobe on the mandible (Fig. 1), the second flagellar segment usually subequal or slightly longer than the first segment, and the precoxal sulcus more or less sculptured.

Our current taxonomic research on the braconid wasps of the subfamily Alysiinae in Iran resulted in the first record of a species of *Adelurola* in this country. The difficulty for the identification of species of *Adelurola* has led to a major revision of this genus. In this work, all the currently recognised species *Adelurola* are re-described and an identification key is provided.

## Material and methods

Sampling in Iran was carried out by sweeping with a standard net in Kermanshah province (western part of Iran) in 2013. Specimens were subsequently prepared using the AXA method (van Achterberg 2009). For terminology of the morphological features and sculpture, measurements and wing venation nomenclature see van Achterberg (1993). Photographs were taken with a Digital Microscope VHX-2000 and with a Nikon® D700 mounted on a Leica® S8APO microscope, with images combined using Helicon Focus® and edited using Adobe Photoshop® imaging system.

Specimens examined are deposited in the collection of the Zoological Institute of the Russian Academy of Sciences, St Petersburg, Russia (ZISP), and in the collection of Naturalis Biodiversity Center, Leiden, the Netherlands (RMNH). Type specimen of *Alysia florimela* Haliday was studied by the third author in the Haliday Collection (Dublin, Ireland) (van Achterberg 1993); the type material of *Neocarpa amplidens* Fischer is missing in the Zoologische Sammlung (Munich, Germany) and its current location is unclear.

## Results

### Genus Adelurola

Taxon classificationAnimaliaHymenopteraBraconidae

Strand, 1928

Adelura Foerster 1863: 267 (not Bonaparte 1854); Shenefelt 1974: 986; van Achterberg 1993: 4; Yu et al. 2012.
Adelurola Strand 1928: 51 (nom. n. for *Adelura* Foerster); Shenefelt 1974: 986; van Achterberg 1993: 4; Yu et al. 2012.
Neocarpa Fischer 1966: 185; Shenefelt 1974: 987; van Achterberg 1993: 4; Yu et al. 2012.

#### Type species.

*Alysia florimela* Haliday, 1838.

#### Diagnosis.

Mandibles distinctly broadened apically, with additional ventral wide fourth denticle (lobe). Eyes glabrous. Second flagellar segment not longer than first segment. Pterostigma short and wide. Radial vein (r) originating almost from its middle. Brachial (first subdiscal) cell of fore wing closed apically. Pronope of mesosoma small or absent. Precoxal sulcus distinctly sculptured. Dorsope of first metasomal tergite distinct. Second tergite smooth. Ovipositor short, shorter than apical height of metasoma.

#### Remarks.

This genus is similar to *Dapsilarthra* but differs from it in having the wide ventral lamelliform lobe on the mandible (Fig. 1) (absent in *Dapsilarthra*), the first flagellar segment not shorter than second segment (shorter in *Dapsilarthra*), precoxal suture sculptured (usually smooth in *Dapsilarthra*), and radial vein (r) arising submedially from pterostigma (usually before pterostigma in *Dapsilarthra*).

The observation of these characters in studied images of the holotype of *Adelurola eurys* Chen & Wu, 1994 showed that this species is better placed under genus *Dapsilarthra* Foerster, 1863 (comb. n.).

#### Hosts.

Cyclorraphous Diptera (Tephritidae and Anthomyiidae).

### Adelurola amplidens

Taxon classificationAnimaliaHymenopteraBraconidae

(Fischer, 1966)

Fig. 2

Neocarpa amplidens Fischer 1966: 85.
Dapsilarthra amplidens : Shenefelt 1974: 987; Wharton et al. 2006: 325.
Adelurola amplidens : van Achterberg 1983: 5; Yu et al. 2012.

#### Material examined.

**Iran**: 2 females, Kermanshah Province, Kermanshah, 16.iv.2013, swept on *Medicago sativa* L. (Z. Sharifi coll.) (ZISP, RMNH); 1 male, Iran, Hormozgan Province, Harsin, 16.iv.2013, swept on *Medicago sativa* L. (S. Sharifi coll.) (ZISP). **Iraq**: 2 females, Baghdad, em. 10.iv.[19]80 and 13.iv.[19]80; L. Jabbar; on Beta vulgaris; ex *Pegomyia hyoscyami*; *Dapsilarthra amplidens* ♀ det. Papp J. 1981” (RMNH).

#### Description.

Female.

Head entirely smooth; in dorsal view twice as wide as median length, 1.5 times as wide as mesoscutum, with rounded temples behind eye. Eye in lateral view 1.5 times as high as wide and 0.8 times as wide as temple medially. POL 1.5 times OD; OOL 3.5 times OD. Face slightly punctate, with scattered short setae, without middle vertical protuberance in upper half, 1.9 times as wide as high; inner margins of eyes subparallel. Clypeus slightly curved ventrally, 1.9 times as wide as high. Mandible widened towards apex, 1.3 times as long as its maximum width. Upper tooth of mandible broadened towards subapex, longer than lower tooth; middle tooth wide basally and narrowed towards apex, rounded apically; lower tooth rounded apically. Antenna thick, 37-segmented. Scape 1.5 times as long as pedicel. First flagellar segment 3.0 times as long as its apical width; second segment 4.4 times as long as its maximum width, about as long as first segment. Third flagellar segment 4.0 times as long as its maximum width. Penultimate segment 2.0 times and apical segment 4.0 times as long as their maximum widths, respectively.

Mesosoma 1.5 times as long as high (lateral view). Mesoscutum smooth, punctate in antero-dorsal area, with numerous scattered setae, about as long as maximum width. Notauli present, punctate, reaching half part of mesoscutum, not reaching with mesoscutal pit. Mesoscutal pit present, elongate. Scutellar sulcus rugose-striate; with median and lateral carinae. Sides of pronotum sculptured. Precoxal suture present, widely rugose-crenulate, not reaching anterior and posterior margins of mesopleuron. Posterior mesopleural furrow crenulated. Propodeum completely rugose-reticulate, with numerous scattered setae. Propodeal spiracle relatively small.

Wings. Length of fore wing 2.3 times its maximum width. Pterostigma cuneate. Marginal cell ending before apex of wing, 2.5 times as long as its maximum width. Vein 3-SR 2.0 times as long as vein 2-SR. Vein SR1 1.8 times as long as vein 3-SR. Second submarginal cell 3.3 times as long as its maximum width. Vein cu-a postfurcal. Subdiscal cell closed, 3.8 times as long as its maximum width. Hind wing 4.6 times as long as its maximum width.

Legs. Hind femur 5.2 times as long as its maximum width. Hind tibia slightly widened towards apex, about 9.7 times as long as its maximum subapical width, 1.1 times as long as hind tarsus. First segment of hind tarsus 1.8 times as long as second segment.

Metasoma slightly compressed laterally. First tergite rugose-reticulate in apical half, without median carinae, slightly widened towards apex, 1.3 times as long as its apical width. Second metasomal tergite smooth. Ovipositor sheath 0.6 times as long as first tergite, 0.4 times as long as hind femur.

Colour. Body brown to dark brown. First metasomal tergite paler than second and third tergites, apical segments dark. Legs yellow, apical part of the tibia and hind tarsus darker than femur. Wings hyaline. Pterostigma brown.

Body length 3.9 mm; fore wing length 4.3 mm.

Male. Body length 3.5 mm; fore wing length 4.0 mm. Eye in lateral view 1.3 times as high as wide. Mandible 1.1 times as long as its maximum width. First flagellar segment 3.2 times as long as its apical width. Hind femur 4.8 times as long as its maximum width. Otherwise differs from female.

Differences of male types (according to original description: Fischer 1966). Fore wing length 4.4 mm. Mandible 1.3 times as long as its maximum width. Antenna 38–39-segmented. First flagellar segment 3.0 times as long as its apical width. Mesoscutum about as long as its maximum width. Hind femur 4.5 times as long as its maximum width.

**Figure 2.**
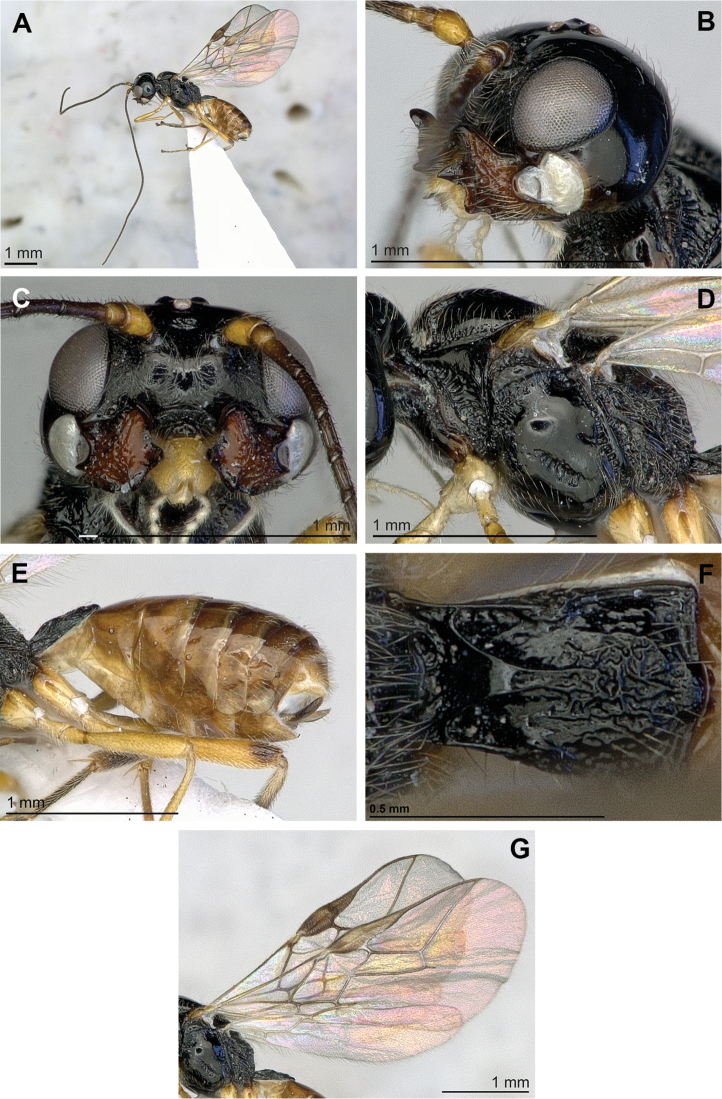
*Adelurola amplidens* (Fischer) (female). **A** Habitus, lateral view **B** Head (lateral view) and mandible **C** Face, front view **D** Mesosoma, lateral view **E** Hind femur, metasoma and ovipositor, lateral view **F** First metasomal tergite **G** Fore and hind wings.

#### Comparative diagnosis.

This species is similar to *Adelurola asiatica* Telenga, 1935 and *Adelurola florimela* (Haliday, 1838). *Adelurola amplidens* differs from *Adelurola asiatica* in having the eye in lateral view 0.8 times as wide as temple medially (1.2 times in *Adelurola asiatica*), marginal cell 2.5 times as long as its maximum width (3.8 times in *Adelurola asiatica*), and precoxal suture not reaching anterior and posterior margins of mesopleuron (reaching anterior and posterior margins in *Adelurola asiatica*). *Adelurola amplidens* differs from *Adelurola florimela* in having the eye in lateral view 0.85 times as wide as temple medially (about 1.2 times in *Adelurola florimela*), first metasomal tergite without median carinae (with median carinae in *Adelurola florimela*), vein 3-SR 2.0 times as long as vein 2-SR (1.1–1.3 times in *Adelurola florimela*), vein SR1 1.8 times as long as veins 3-SR (2.2–2.6 times in *Adelurola florimela*), and marginal cell 2.5 times as long as its maximum width (2.8–3.2 times in *Adelurola florimela*).

#### Distribution.

Iraq, Iran (new record).

### Adelurola asiatica

Taxon classificationAnimaliaHymenopteraBraconidae

Telenga, 1935

Fig. 3

Adelurola asiatica Telenga 1935: 186; van Achterberg 1983: 6; Tobias 1986: 236; Yu et al. 2012.
Dapsilarthra asiatica : Königsmann 1959: 599; Fischer 1970: 11; 1971: 78; Shenefelt 1974: 987.

#### Type material.

Holotype: female (nearly entirely destroyed; head, antennae, all wings, metasoma, right fore and hind legs missing), **Uzbekistan**, silver circle, “Yargak (Jargak), Khatvirg. r. (?), 24.iv.[19]28, L. Zimin” (ZISP).

#### Additional material.

**Kyrgyzstan**: 1 female, “20 km S of Toktogul, gorge of Karasu River, forest, 25.vii.1982, S. Belokobylskij coll.” (ZISP). **Turkmenistan**: 1 female, “between Sumbar and Chandyr Rivers, Monzhukly Mountain Range, Kara-Yantam gorge, 2 km E of Karakel Aul, 28.iv.1993, V. Perepechaenko coll.” (ZISP).

#### Description.

Female.

Head entirely smooth; in dorsal view 1.8 times as wide as median length, 1.45 times as wide as mesoscutum, with convex rounded temples behind eye. Eye in lateral view 1.35 times as high as wide and 0.9 times as wide as temple medially. POL 0.8 times OD; OOL 2.8 times OD. Face smooth, with very fine reticulation, with scattered short setae in lateral areas, with low middle vertical protuberance, 2.1 times as wide as high; inner margins of eyes subparallel. Clypeus slightly convex ventrally, about twice as wide as high. Mandible broadened towards subapex, 0.9 times as long as its maximum width. Upper tooth of mandible broadened sideward, much longer than lower tooth; middle tooth wide basally and narrowed towards apex, rounded apically; lower tooth short, rounded apically. Antenna rather slender, 37-segmented. Scape 1.15 times as long as pedicel. First flagellar segment 3.1 times as long as its apical width; second segment 3.7 times as long as its maximum width, 1.2 times as long as first segment. Third flagellar segment about 3.0 times as long as its maximum width. Penultimate segment 2.2 times and apical segment 4.2 times as long as their maximum widths, respectively.

Mesosoma 1.5 times as long as high (lateral view). Mesoscutum smooth, punctate and densely setose in anterior part, 0.9 times as long as maximum width. Notauli coarsely crenulate, present in anterior half of mesoscutum, not reaching with mesoscutal pit. Mesoscutal pit present, very long, sparsely crenulate. Scutellar sulcus distinctly crenulate, with median carina but without lateral carinae. Sides of pronotum smooth in anterodorsal area, mainly rugose-reticulate. Precoxal suture present, wide, reaching anterior margin of mesopleuron but absent posteriorly. Posterior mesopleural furrow sparsely and widely crenulate below and shortly and densely crenulate in upper half. Propodeum completely rugose-reticulate, with numerous scattered setae. Propodeal spiracle relatively small.

Wings. Length of fore wing 2.3 times its maximum width. Pterostigma cuneate. Marginal cell distinctly shortened, reaching distinctly before apex of wing, 2.8 times as long as its maximum width. Vein 3-SR 1.7 times as long as vein 2-SR. Vein SR1 1.9 times as long as vein 3-SR. Second submarginal cell 3.3 times as long as maximum width. Vein cu-a distinctly postfurcal. Subdiscal cell closed, 2.3 times as long as its maximum width. Hind wing 4.0 times as long as its maximum width.

Legs. Hind femur 4.7 times as long as its maximum width. Hind tibia slightly widened towards apex, about 10.0 times as long as its maximum subapical width, as long as hind tarsus. First segment of hind tarsus 1.9 times as long as second segment.

Metasoma compressed laterally. First tergite completely and densely rugose-reticulate, without median carinae, slightly widened towards apex, 1.4 times as long as its apical width. Second metasomal tergite smooth. Ovipositor sheath 0.6 times as long as first tergite, 0.3 times as long as hind femur.

Colour. Body reddish brown to dark brown or black. Metasoma medially light reddish brown, apical segments dark brown. Legs mainly yellow, hind femur and tibia dark. Wings very faintly infuscate. Pterostigma brown.

Body length 3.2 mm; fore wing length 3.0 mm.

Variation. Body length 3.4 mm; fore wing length 3.3 mm. Mandible 0.8 times as long as its maximum width; middle teeth distinctly reduced. Antenna 39-segmented. First flagellar segment 3.3 times as long as its apical width; second segment 3.4 times as long as its maximum width, 1.1 times as long as first segment. Mesoscutum about as long as its maximum width. Length of fore wing 2.3 times its maximum width. Marginal cell 3.0 times as long as its maximum width. Vein 3-SR 1.55 times as long as vein 2-SR. Vein SR1 2.0 times as long as veins 3-SR. Second submarginal cell 3.4 times as long as its maximum width. Hind wing 4.3 times as long as its maximum width. Hind femur 5.3 times as long as its maximum width. First metasomal tergite 1.5 times as long as its apical width.

Male. Unknown.

**Figure 3.**
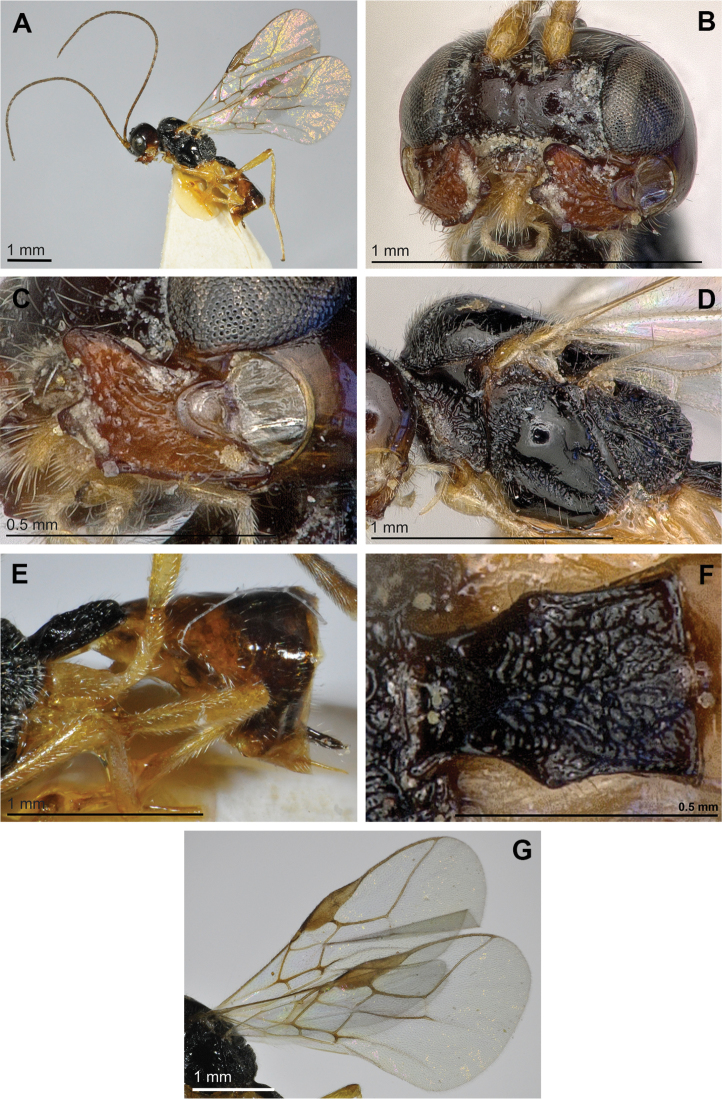
*Adelurola asiatica* Telenga (female). **A** Habitus, lateral view **B** Face, front view **C** Mandible **D** Mesosoma, lateral view **E** Metasoma and ovipositor, lateral view **F** First metasomal tergite **G** Fore and hind wings.

#### Diagnosis.

This species is similar to *Adelurola amplidens* (Fischer, 1966) and *Adelurola florimela* (Haliday, 1838). *Adelurola asiatica* differs from *Adelurola florimela* by having the mandible 1.25 times as long as its maximum width (1.0 times in *Adelurola florimela*), first metasomal tergite without median carinae (with median carinae in *Adelurola florimela*), vein 3-SR 1.9 times as long as vein 2-SR (1.2 times in *Adelurola florimela*), vein SR1 1.8 times as long as vein 3-SR (2.4 times in *Adelurola florimela*), and precoxal suture reaching anterior and posterior margins of mesopleuron (not reaching anterior and posterior margins in *Adelurola florimela*). Differences between *Adelurola asiatica* and *Adelurola amplidens* are in the re-description of the latter species.

#### Distribution.

Uzbekistan, Turkmenistan, Kyrgyzstan (new record).

### Adelurola florimela

Taxon classificationAnimaliaHymenopteraBraconidae

(Haliday, 1838)

Fig. 4

Alysia florimela Haliday 1838: 239; 1839: 25.
Adelura florimela : Foerster 1863: 267; Marshall 1894: 420; Dalla Torre 1901: 38; Lyle 1933: 74; Morley 1933: 183; Stelfox 1941: 2.
Alysia (Adelura) florimela : Thomson 1895: 2287.
Dapsilarthra florimela : Kloet and Hincks 1945: 239; Königsmann 1959: 589; Fischer 1970: 13; Shenefelt 1974: 988; van Achterberg 1997: 40; Wharton et al. 2006: 325.
Adelurola florimela : van Achterberg 1983: 5; Gurasashvili 1983: 784; Tobias 1986: 236; Belokobylskij 1998: 284; 2003: 356; Yu et al. 2012; Broad et al. 2012: 7; Riedel and Hansen 2014: 148.
Phaenocarpa multiarticulata Marshall 1898: 245.
Dapsilarthra multiarticulata : Shenefelt 1974: 989.
Dapsilarthra pentapleuroides Fischer 1971: 85 (as synonym of *Adelurola multiarticulata*).

#### Material examined.

**Germany**: 1 female, “*Adelura florimela* Hal. ♀”, “Schmiedeknecht dt.”; 2 males, without geographical labels, from Schmiedeknecht Collection. **Latvia**: 1 female, Valmier Region, Draudziba, ex larva of *Pegomyia hyoscyami* (Panzer) on beet, 6.vii.1962, V. Ozolinsh coll. (ZISP); 1 female, same label, but 14.07.1962 (ZISP); 1 female, same label, but 21.viii.1962 (ZISP). **Russia**: 1 female, 2 males, Leningrad Province, Kingisepp, 20 and 22.v.1904, Vinogradov-Nikitin coll. (ZISP); 1 female, Yaroslavl’ Province, Bykovo, 25.v.1891, N. Kokuev coll. (ZISP); 1 female, Yamalo-Nenetsk Autonomous Region, Krasnosel’kup, Taz River, terrace, 15–17.viii.1992, D. Kasparyan coll. (ZISP); 1 male, PrimorskiyTerritory, 12 km S Khorol’, forest, 4.vi.1979, S. Belokobylskij coll. (ZISP); 1 male, Primorskiy Territory, 8 km from Brovnichi, Serebryanoe, 9.vi.1978, A. Kupyanskaya coll. (ZISP); 1 male, Primorskiy Territory, Vladivostok, Sedanka, 3.vi.1978, S. Belokobylskij coll. (ZISP); 1 female, Primorskiy Territory, 30 km SE Ussuriysk, Ussuriysk Nature Reserve forest, 10–11.vi.1993, S. Belokobylskij coll. (ZISP); 1 female, Sakhalin Island, Yuzhno-Sakhalinsk environs, Chekhov Mountain, 900 m, 28.vii.1988, A. Kotenko coll. (ZISP). **Georgia**: 1 female, Kazbegi, 2300 m, meadow, 16.viii.1982, M. Gurasashvili coll. (ZISP); for material in RMNH from the Netherlands, Germany and Bulgaria, see van Achterberg (1983).

#### Description.

Female.

Head entirely smooth; in dorsal view 2.0 times as wide as median length, 1.5 times as wide as mesoscutum, with not convex rounded temples behind eye. Eye in lateral view 1.3 times as high as wide and 1.15 times as wide as temple medially. POL about as long as OD; OOL 3.0 times OD. Face rugulose medially, with scattered setae, with distinct and complete middle prominence, 2.0 times as wide as high; inner margins of eyes subparallel. Clypeus slightly curved ventrally, 3.0 times as wide as high. Mandible broadened towards subapex, 1.2 times as long as its maximum width, rugose. Upper tooth of mandible broadened sideward, distinctly longer than lower tooth; middle tooth wide basally and narrowed towards apex, pointed apically; lower tooth rounded apically. Antenna rather slender, 45-segmented. Scape 1.25 times as long as pedicel. First flagellar segment 3.3 times as long as its apical width; second segment 3.8 times as long as its maximum width, 1.1 times as long as first segment. Third flagellar segment 3.5 times as long as its maximum width. Penultimate segment about 2.0 times and apical segment 3.3 times as long as their maximum widths, respectively.

Mesosoma 1.4 times as long as high (lateral view). Mesoscutum entirely smooth, with dense setae along notauli and scattered setae laterally, as long as its maximum width. Notauli present in anterior half and absent in posterior half, crenulate. Mesoscutal pit present, short, elongate. Scutellar sulcus finely and sparsely rugulose, with distinct median carina but without lateral carinae. Sides of pronotum mainly smooth. Precoxal suture rather wide and rugulose, reaching anterior margin of mesopleuron, but absent posteriorly. Posterior mesopleural furrow completely crenulate. Propodeum completely rugose-reticulate. Propodeal spiracle small.

Wings. Length of fore wing 2.6 times its maximum width. Pterostigma cuneate. Marginal cell just not reaching apex of wing, 3.2 times as long as its maximum width. Vein 3-SR 1.3 times as long as vein 2-SR. Vein SR1 2.2 times as long as veins 3-SR. Second submarginal cell 3.6 times as long as its maximum width. Vein cu-a distinctly postfurcal. Subdiscal cell closed, 2.5 times as long as its maximum width. Hind wing 4.3 times as long as its maximum width.

Legs. Hind femur about 5.0 times as long as its maximum width. Hind tibia slightly widened towards apex, about 10.0 times as long as its maximum subapical width, 0.9 times as long as hind tarsus. First segment of hind tarsus 2.0 times as long as second segment.

Metasoma depressed dorso-ventrally. First tergite completely rugose-reticulate with median carina, hardly widened towards apex (subparallel), 1.6 times as long as its apical width. Second metasomal tergite smooth. Ovipositor sheath 0.3 times as long as first tergite, 0.2 times as long as hind femur.

Colour. Body brown to dark reddish brown. Second metasomal tergite reddish brown, paler than first and apical tergites. Legs yellowish brown, hind tibia apically and most part of hind tarsus distinctly infuscate. Wings almost hyaline. Pterostigma brown.

Body length 3.4 mm; fore wing length 3.9 mm.

Variation. Body length 3.1–4.2 mm; fore wing length 3.5–4.3 mm. Antenna 43–49-segmented. First flagellar segment 3.0–3.4 times as long as its apical width; second segment 3.7–4.2 times as long as its maximum width, 1.10–1.15 times as long as first segment. Third flagellar segment 3.5–4.0 times as long as its maximum width. Marginal cell of fore wing 2.8–3.1 times as long as its maximum width. Vein 3-SR 1.1–1.3 times as long as vein 2-SR. Vein SR1 2.2–2.6 times as long as vein 3-SR. Second submarginal cell 3.0–3.5 times as long as its maximum width. Subdiscal cell 2.1–3.0 times as long as its maximum width. Hind femur 4.7–5.0 times as long as its maximum width. First tergite 1.3–1.6 times as long as its apical width. Ovipositor sheath 0.3–0.6 times as long as first tergite, 0.2–0.4 times as long as hind femur.

Male. Body length 3.2–4.0 mm; fore wing length 3.6–4.6 mm. Mandible often brown to dark brown. Veins of fore wing more or less widened; pterostigma distinctly thickened and completely black or dark brown. Hind femur 5.0 times as long as its maximum width. First metasomal tergite narrow, 1.6–1.9 times as long as its apical width.

**Figure 4.**
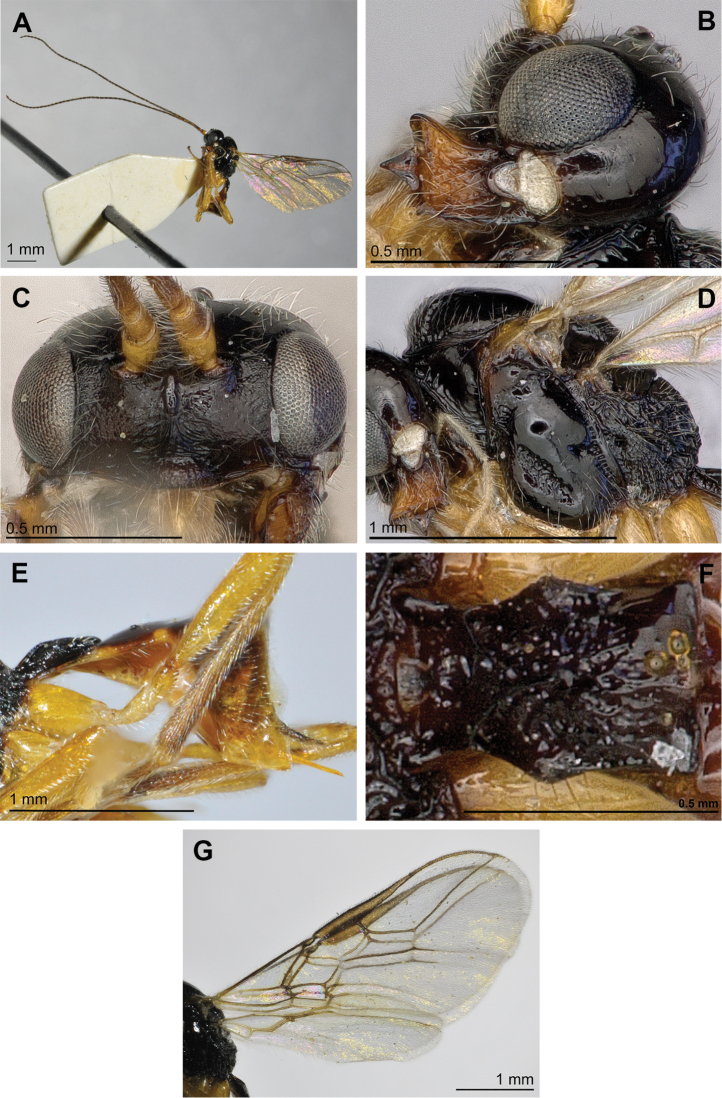
*Adelurola florimela* (Haliday) (female). **A** Habitus, lateral view **B** Head (lateral view) and mandible **C** Face, front view **D** Mesosoma, lateral view **E** Metasoma and ovipositor, lateral view **F** First metasomal tergite **G** Fore and hind wings.

#### Diagnosis.

*Adelurola florimela* (Haliday, 1838) differs from *Adelurola amplidens* (Fischer, 1966), *Adelurola asiatica* Telenga, 1935 and *Adelurola kamtshatica* Belokobylskij, 1998 by the features listed in the diagnoses of each of these species, as listed above.

#### Hosts.

*Acidia cognata* (Wiedemann, 1817) (Tephritidae), *Pegomya hyoscyami* (Panzer, 1809), *Pegomya nigritarsis* (Zetterstedt, 1838) and *Pegomya solennis* (Meigen, 1826) (Anthomyiidae) (van Achterberg 1983; Belokobylskij 1998; Yu et al. 2012).

#### Distribution.

Austria, former Czechoslovakia, Finland, Georgia, Germany, Hungary, Ireland, Italy, Japan, Latvia, Lithuania, Netherlands, Poland, Russia, Slovenia, Spain, Sweden, Switzerland, United Kingdom, former Yugoslavia.

### Adelurola kamtshatica

Taxon classificationAnimaliaHymenopteraBraconidae

Belokobylskij, 1998

Fig. 5

Adelurola kamtshatica Belokobylskij 1998: 285; Yu et al. 2012.

#### Type material.

Holotype: female, Kamchatka, saddle of Avacha and Koryaka Volcanoes, 1000 m, mountain tundra, 27.vii.1985, S. Belokobylskij coll. (ZISP); 1 female (paratype), same label, but 26.vii.1985 (ZISP).

#### Description.

Female.

Head entirely smooth; in dorsal view 2.0 times as wide as median length, 1.5 times as wide as mesoscutum, with rounded temples behind eye. Eye in lateral view 1.2 times as high as wide and 0.9 times as wide as temple medially. POL as long as OD; OOL 4.3 times OD. Face faintly rugulose-striate, with rather dense setae, with complete middle prominence, 2.4 times as wide as high; inner margins of eyes subparallel. Clypeus distinctly curved ventrally, 2.0 times as wide as high. Mandible not strongly broadened towards subapex,1.3 times as long as its maximum width, rugose. Upper tooth of mandible broadened sideward, distinctly longer than lower tooth; middle tooth wide basally and strongly narrowed towards apex, pointed apically; lower tooth rounded apically. Antenna slender, 45-segmented. Scape 1.9 times as long as pedicel. First flagellar segment 2.8 times as long as its apical width; second segment 3.6 times as long as its maximum width, 1.25 times as long as first segment. Third flagellar segment 3.1 times as long as its maximum width. Penultimate segment 2.4 times and apical segment 3.0 times as long as their maximum widths, respectively.

Mesosoma 1.4 times as long as high (lateral view). Mesoscutum smooth, its upper part with dense setae latero-anteriorly, 1.1 times as long as maximum width. Notauli absent in posterior half. Mesoscutal pit present, elongate. Scutellar sulcus smooth, without median and lateral carinae. Sides of pronotum mainly smooth. Precoxal suture present, not reaching anterior and posterior margins of mesopleuron, slightly crenulate. Posterior mesopleural furrow completely and shortly crenulate. Propodeum completely rugose-reticulate, with numerous scattered setae. Propodeal spiracle small.

Wings. Length of fore wing 2.2 times its maximum width. Pterostigma cuneate. Marginal cell reaching just before apex of wing, 3.5 times as long as its maximum width. Vein 3-SR 1.3 times as long as vein 2-SR. Vein SR1 2.6 times as long as vein 3-SR. Second submarginal cell 3.1 times as long as its maximum width. Vein cu-a postfurcal. Subdiscal cell closed, 2.6 times as long as its maximum width. Hind wing 4.3 times as long as its maximum width.

Legs. Hind femur 4.7 times as long as its maximum width. Hind tibia slightly widened towards apex, about 10.0 times as long as its maximum subapical width, as long as hind tarsus. First segment of hind tarsus 1.9 times as long as second segment.

Metasoma compressed laterally. First tergite slightly rugose-reticulate in apical half, with several striae, with median carinae, not widened towards apex (parallel subparallel), 1.6 times as long as its apical width. Second metasomal tergite smooth. Ovipositor sheath 0.7 times as long as first tergite, 0.4 times as long as hind femur.

Colour. Body dark brown dark reddish brown. Second and third metasomal tergites light reddish brown, apical tergites faintly pale. Legs yellowish brown, hind femur apically, hind tibia in apical half and most part of hind tarsus distinctly infuscate. Wings hyaline. Pterostigma brown basally and pale brown apically.

Body length 2.9 mm; fore wing length 3.5 mm.

Variation. Fore wing length 3.4 mm. Head in dorsal view 1.9 times as wide as median length POL 0.8 times as long as OD. Face about twice as wide as high. Clypeus 2.5 times as wide as high. Mesosoma 1.5 times as long as high (lateral view). Length of fore wing 2.4 times its maximum width. Vein 3-SR 1.4 times as long as vein 2-SR. Vein SR1 twice as long as veins 3-SR. Second submarginal cell 3.4 times as long as maximum width. Subdiscal cell 2.3 times as long as its maximum width. Hind femur 4.6 times as long as its maximum width. First tergite 1.5 times as long as its apical width. Ovipositor sheath 0.8 times as long as first tergite.

Male. Unknown.

**Figure 5.**
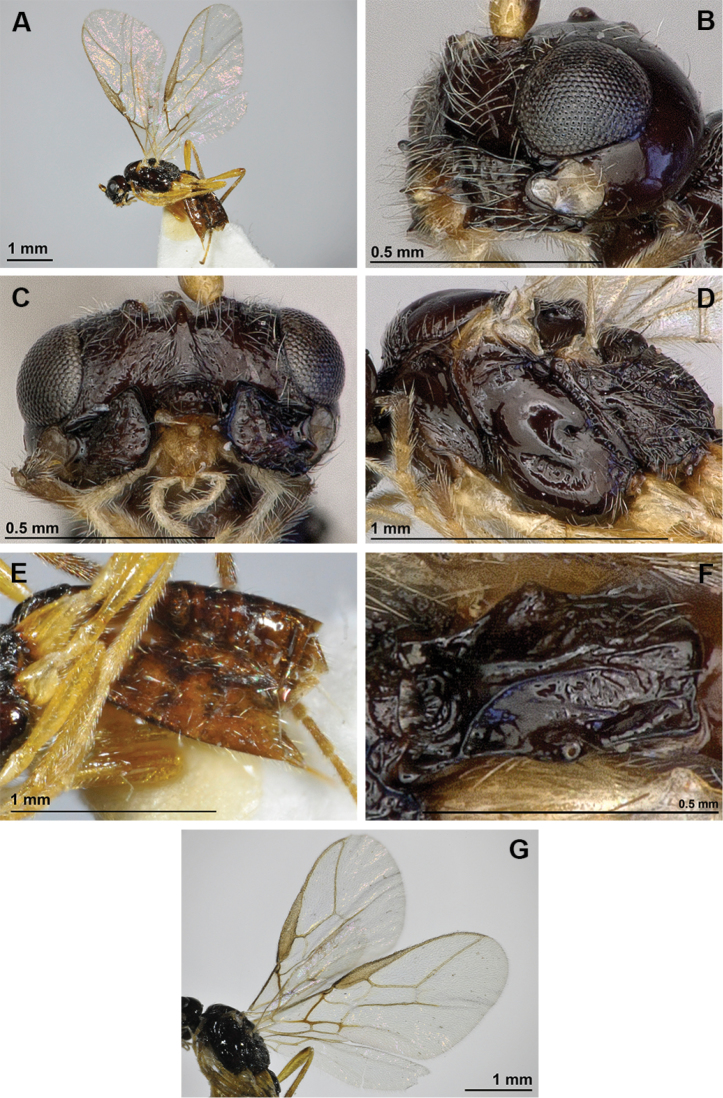
*Adelurola kamtshatica* Belokobylskij (female, paratype). **A** Habitus, lateral view **B** Head (lateral view) and mandible **C** Face in front view **D** Mesosoma, lateral view **E** Metasoma and ovipositor, lateral view **F** First metasomal tergite **G** Fore and hind wings.

#### Diagnosis.

This species is similar to *Adelurola florimela* (Haliday, 1838), but differs from it in having the scutellar sulcus smooth and without complete median carina (sculptured and with complete median carina in *Adelurola florimela*), first flagellar segment shorter (longer in *Adelurola florimela*), mandible slightly widened towards apex and its upper tooth smaller and less protruding upwards (distinctly widened and with large upper tooth in *Adelurola florimela*), precoxal suture finely and narrow rugulose (distinctly and widely rugose in *Adelurola florimela*), pterostigma paler, pale brown (darker, brown in *Adelurola florimela*).

#### Distribution.

Russia (Far East).

### Key to the world species of *Adelurola*

**Table:** 

1	Mandible slightly widened towards apex (Fig. 5B), upper tooth smaller and less distant up (Fig. 5B). Prescutellar sulcus without median carina and almost smooth. Precoxal suture finely and narrowly rugulose (Fig. 5D). Body length 2.8–2.9 mm	***Adelurola kamtshatica* Belokobylskij**
–	Mandible distinctly widened towards apex (Figs 2B, 3C, 4B), upper tooth larger and more distant up (Figs 2B, 3B, 4B). Prescutellar sulcus with median carina and sculptured. Precoxal suture distinctly and widely rugose (Figs 2D, 3D, 4D)	**2**
2(1)	First flagellar segment 0.8 times as long as second segment. Precoxal suture reaching anterior and usually posterior margins of mesopleuron (Fig. 3D). Third mandibular tooth small or very small (Fig. 3C). Body length 3.2–3.4 mm	***Adelurola asiatica* Telenga**
–	First flagellar segment 0.95–1.05 times as long as second segment. Precoxal suture short, not reaching anterior and posterior margins of mesopleuron or reaching only anterior margin (Fig. 2D, 4D). Third mandibular tooth rather large (Figs 2B, 4B)	**3**
3(2)	Eye in lateral view 1.2 times as wide as temple medially (Fig. 4B). Metasoma mainly depressed dorso-ventrally (Fig. 4E). Marginal cell almost reaching apex of wing, 2.8–3.2 times as long as its maximum width (Fig. 4F). Vein 3-SR 1.1–1.3 times as long as vein 2-SR (Fig. 4F). Vein SR1 2.2–2.6 times as long as vein 3-SR (Fig. 4F). Body length 3.1–4.2 mm
***A. florimela* (Haliday)**
–	Eye in lateral view 0.8 times as wide as temple medially (Fig. 2B). Metasoma more or less compressed laterally (Fig. 2E). Marginal cell remaining far from apex of wing, 2.5 times as long as its maximum width (Fig. 2F). Vein 3-SR 2.0 times as long as vein 2-SR (Fig. 2F). Vein SR1 1.8 times as long as vein 3-SR (Fig. 2F). Body length 3.5–3.9 mm	***Adelurola amplidens* (Fischer)**

### Excluded species

#### Dapsilarthra eurys

Taxon classificationAnimaliaHymenopteraBraconidae

(Chen & Wu, 1994) comb. n.

Adelurola eurys Chen and Wu 1994: 19, 154; Yu et al. 2012.

##### Type material.

Holotype: female (studied the images), China, “Xianfengling, Mt. Wuyi, Fujian, 2.viii.1986, Liu Minghui” (Beneficial Insects Institute, Fujian Agricultural University, Fuzhou, China).

##### Remarks.

After examining of the holotype images of *Adelurola eurys* we surely considered that this species actually belongs to the genus *Dapsilarthra* by the absence of a ventral lamelliform lobe on mandible and elongated the first flagellar segment of antenna.

## Discussion

*Adelurola* is exclusively Palaearctic small genus of parasitoid wasps of subfamily Alysiinae closely related to *Dapsilarthra*. If *Adelurola florimela* (Haliday) is very widely distributed in the Palaearctic Region (from U.K. till Russian Far East), then other three species have local Asian distribution on the territories of Middle East [*Adelurola amplidens* (Fischer)], Central Asia (*Adelurola asiatica* Telenga) and Kamchatka Peninsula (*Adelurola kamtshatica* Belokobylskij). Additionally this paper includes the first records of *Adelurola* species, *Adelurola amplidens* and *Adelurola asiatica* Telenga, from Iran and Kyrgyzstan.

Unfortunately, the hosts of *Adelurola* taxa are unknown yet. However published in this paper information is valuable one owing to significant role of many Alysiinae taxa in the regulation of the natural dipterans populations mainly from families Anthomyiidae and Tephritidae.

## Supplementary Material

XML Treatment for Adelurola

XML Treatment for Adelurola amplidens

XML Treatment for Adelurola asiatica

XML Treatment for Adelurola florimela

XML Treatment for Adelurola kamtshatica

XML Treatment for Dapsilarthra eurys

## References

[B1] Achterberg C van (1983) Revisionary notes on the genera *Dapsilarthra* auct. and *Mesocrina* Foerster (Hymenoptera, Braconidae, Alysiinae). Tijdschrift voor Entomologie 126: 1–24.

[B2] Achterberg C van (1993) Illustrated key to the subfamilies of the Braconidae (Hymenoptera: Ichneumonoidea). Zoologische Verhandelingen Leiden 283: 1–189.

[B3] Achterberg C van (1997) Revision of the Haliday collection of Braconidae (Hymenoptera). Zoologische Verhandelingen Leiden 314: 1–115.

[B4] Achterberg C van (2009) Can Townes type Malaise traps be improved? Some recent developments. Entomologische Berichten Amsterdam 69(4): 129–135.

[B5] Belokobylskij SA (1998) 9. Alysiinae (Alysiini). In: Ler PA Key to the insects of Russian Far East. Vol. 4. Neuropteroidea, Mecoptera, Hymenoptera. Pt 3. Dal’nauka, Vladivostok, 162–298. [In Russian]

[B6] Belokobylskij SA Taeger A Achterberg van C Haeselbarth E Riedel M (2003) Checklist of the Braconidae (Hymenoptera) of Germany. Beiträge fur Entomologie 53(2): 341–435.

[B7] Broad GR Shaw MR Godfray HCJ (2012) Checklist of British and Irish Braconidae (Hymenoptera). http://www.nhm.ac.uk/resources-rx/files/braconidae-checklist-for-web-34139.pdf [accessed 17 September 2014]

[B8] Chen J Wu Z (1994) The Alysiini of China (Hymenoptera, Braconidae, Alysiinae). China Agricultural press, Fuzhou, China, 218 pp [In Chinese]

[B9] Dalla Torre CG de (1901) Catalogus Hymenopterorum hucusque descriptorum systematicus et synonymicus. Vol. III: Trigonalidae, Megalyridae, Stephanidae, Ichneumonidae, Agriotypidae, Evaniidae, Pelecinidae. Guilelmi Engelmann 1901: 1–544.

[B10] Fischer M (1966) Studien uber Alysiinae (Hymenoptera, Braconidae). Annalen des Naturhistorischen Museums in Wien 69: 177–205.

[B11] Fischer M (1970) Die Alysiini der Steiermark (Hymenoptera, Braconidae). Mitteilungen der Abteilung für Zoologie am Landesmuseum Joanneum 34: 1–44.

[B12] Fischer M (1971) Untersuchungen über die europäischen Alysiini mit besonderer Berücksichtigung der Fauna Niederösterreichs (Hymenoptera, Braconidae). Polskie Pismo Entomologiczne 41(1): 19–160.

[B13] Foerster A (1863) Synopsis der Familien und Gattungen der Braconiden. Verhandlungen des Naturhistorischen Vereins der Preussischen Rheinlande und Westfalens 19: 225–288.

[B14] Haliday AH (1838) Essay on parasitic Hymenoptera. Entomological Magazine 5(3): 209–249.

[B15] Haliday AH (1839) Hymenoptera Brittanica: *Alysia*. Ballerie, London, 28 pp.

[B16] Kloet GS Hincks WD (1945) A check list of British insects. Kloet & Hincks, Stockport, 483 pp.

[B17] Königsmann E (1959) Revision der paläarktischen Arten der Gattung *Dapsilarthra*. 1. Beitrag zur systematischen Bearbeitung der Alysiinae (Hymenoptera: Braconidae). Beitrage zur Entomologie 9(5-6): 580–608.

[B18] Lyle GT (1933) A catalogue of British Braconidae. Transactions of the Royal Entomological Society of London 81: 67–74. doi: 10.1111/j.1365-2311.1933.tb00399.x

[B19] Marshall TA (1894) A monograph of the British Braconidae Part V. Transactions of the Royal Entomological Society of London 1894: 497–534. doi: 10.1111/j.1365-2311.1894.tb02098.x

[B20] Marshall TA (1898) Description de Braconides. Bulletin du Museum National d’Histoire Naturelle, 4: 369–371.

[B21] Morley C (1933) Notes on Braconidae XIV: Alysiides. Entomologist 66: 183–185.

[B22] Riedel M Hansen LO (2014) Braconidae (Hymenoptera) of Norway, Part II. Norwegian Journal of Entomology 61: 147–159.

[B23] Shenefelt RD (1974) Braconidae 7. Alysiinae. Hymenopterum Catalogus. Pars 11: 937–1113.

[B24] Stelfox AW (1941) Descriptions of five new species of Alysiidae (Hymenoptera) and notes on some others. Proceedings of the Royal Irish Academy 47(B): 1–16.

[B25] Strand E (1928) Miscellanea nomenclatorica zoologica et palaeontologica. I-II. Archiv für Naturgeschichte (A) 92(8): 30–75.

[B26] Telenga NA (1935) Beiträge zur Kenntnis der Tribus Alysiini (Braconidae, Hymenoptera) aus USSR. Konowia 14: 186–190.

[B27] Thomson CG (1895) LII. Bidrag till Braconidernas Kannedom. Opuscula Entomologica 20: 2141–2339.

[B28] Tobias VI (1986) Subfam. Alysiinae. In: Medvedev GS (Ed.) Opredelitel nasekomykh Evropeiskoi chasti SSSR [Key to insects of the European part of the USSR] 3(5). Nauka, Leningrad, 100–231. [In Russian]

[B29] Wharton R (1980) Review of the Nearctic Alysiini (Hymenoptera, Braconidae): with discussion of generic relationships within the tribe. Univ of California Press 88: 1–112.

[B30] Wharton RA Yoder MJ Gillespie JJ Patton JC Honeycutt RL (2006) Relationships of *Exodontiella*, a non-alysiine, exodont member of the family Braconidae (Insecta, Hymenoptera). Zoologica Scripta 35(4): 323–340. doi: 10.1111/j.1463-6409.2006.00236.x

[B31] Yu DS Achterberg C van Horstmann K (2012) Taxapad 2012, Ichneumonoidea 2011. Database on flash-drive. Ottawa, Ontario, Canada.

